# The efficacy of intralesional vitamin D3 injection in the treatment of cutaneous warts: A clinical therapeutic trial study

**DOI:** 10.1111/srt.13442

**Published:** 2023-08-16

**Authors:** Haider Al‐Sabak, Mohammed Al‐Hattab, Marwah Al‐Rammahi, Muhsin Al‐Dhalimi

**Affiliations:** ^1^ Head of Dermatology and Laser Department, College of Medicine University of Kufa Najaf Iraq; ^2^ Department of Dermatology University of Babylon College of Medicine Babylon Iraq; ^3^ Department of Dermatology Al‐Sadr Medical City Najaf Iraq; ^4^ Dermatology and Laser Department, College of Medicine University of Kufa Najaf Iraq

**Keywords:** vitamin D, wart

## Abstract

**Background:**

The human papillomavirus that causes warts is usually harmless, and it can infect any part of the skin or mucous membranes. Despite the availability of several treatments, warts often return, and scarring, pigmentation changes, and recurrence are all possible side effects.

**Aim:**

Intralesional vitamin D3 was employed as an immunotherapy for cutaneous warts in the current investigation.

**Materials and Methods:**

In Al‐Sadr Medical City in the city of Al‐Najaf Al‐Ashraf, a skin clinic conducted a therapeutic clinical experiment. A total of 204 cutaneous warts were examined in 40 patients (14 men and 26 females). Vitamin D3 solution of the dose (600 000 IU) was injected into the lesions' bases, with 0.2 mL per patient. Each session could only inject up to five warts. The injections were given every 2 weeks for a total of four times. Instructing patients to forego the use of any topical or oral medication was also a part of the protocol. Each patient was evaluated for recurrence risk before each therapy and then again 6 months later.

**Result and Discussion:**

There was a wide range of patient ages in this study, from 20 months to 52 years. About 65% of the cases included females. When looking at the many kinds of warts, the most prevalent was the common wart (71.6%). After four treatments, a positive response was considered to have occurred when all lesions had disappeared, a partial response when more than half of the lesions had disappeared, and no reaction when less than half of the lesions had disappeared. The final tally was 81.9% for those who responded in whole, 11.3% for those who responded partially, and 6.9% for those who did not respond at all. Next‐session complete response rates were 12.7%, 29.9%, 54.9%, and 81.9%, respectively. Thirteen people experienced adverse symptoms, most noticeably minor swelling and itching. Within 6 months of follow‐up, warts had completely disappeared for all patients with a partial or modest response except one who had no reaction.

**Conclusion:**

Vitamin D3 administered intralesional is an effective and low‐cost treatment for cutaneous warts.

## 1 INTRODUCTION

Warts on the skin are benign tumors caused by keratinocyte infection with the human papillomavirus (HPV).^1^ HPV infection is widespread and usually causes no symptoms, but pathological HPV infection can be very dangerous and difficult to treat.^2^ HPV‐related warts and cancers may be particularly troublesome for patients with chronic immune suppression, such as those with hereditary immunodeficiency or who have just received a transplant and are on large doses of immune suppression medication.^3^ Subclinical, latent, and clinical HPV infection all exist. As a result, subclinical abnormalities are only detectable through the use of diagnostic aids (e.g., acetic acid soaking). Clinically normal skin may harbor the HPV virus or another viral genome, a condition known as latent infection. Gross examination of a patient reveals lesions.^4^ PVs have a high rate of causing chronic infections, suggesting that HPVs have evolved strategies to evade immune surveillance.^5^ The cell‐mediated immunity is the primary defense against warts. Stimulating the immune system is the best way to get rid of warts, whereas chronic problems of cell‐mediated immunity increase the severity and prevalence of verruca and the risk of cancers due to HPV.^6^ Resolving plane warts have been studied, and the results demonstrate an infiltration of Langerhans cells, phagocytes, and lymphocytes, including suppressor and helper T cells, as well as satellite‐cell necrosis, all of which are consistent with a cell‐mediated assault.^7^

Nearly two thirds of cutaneous warts spontaneously retreat within 2 years, and the sites of numerous infections commonly regress simultaneously, despite the relative success of papillomaviruses in evading the immune response.^8^ The clinical appearance of HPV‐related skin lesions varies widely. It was assumed that the host, the age of the lesions, and the anatomical location of the lesions all played a role in this previously.^9^ Exophytic, hyperkeratotic, dome‐shaped papules, or plaques are the typical appearance of common warts. Although these warts can appear anywhere on the skin's surface, they are most frequently found on the palms and fingers, as well as other areas that are prone to damage, such as the elbows and knees. A linear pattern of warts may develop after autoinoculation via scratching.^10^ There is currently no reliable, effective, or virucidal method of treating cutaneous warts by either destructive or medicinal methods.^11^

Salicylic acid induces an inflammatory reaction and has a keratolytic impact, both of which serve to reduce the thickness of the wart. This was confirmed in a placebo‐controlled analysis of six cases; patients given the real treatment were 75% successful, whereas those given the placebo were 48% successful.^12^ Using a TCA 80%–90% solution in the office is a popular therapy that has devastating local effects on tissue. TCA can be used throughout pregnancy and has no systemic harmful effects, but scarring may occur after cutaneous injury.

Podophyllin, a resin extracted from plants, is loaded with many harmful chemicals in unknown proportions. Podophyllotoxin is the most potent of these chemicals. Mucosal surfaces, as opposed to keratinized ones, are more vulnerable to their impacts. They have an antimitotic impact by interfering with the development of the spindle on that chromosomes align during mitosis.^13^ A 5% of 5‐FU solution applied under occlusion once daily per a month is more effective than a placebo; however, periungual administration can lead to onycholysis.^14^ Even though hyperpigmentation, erosion, and erythema may be manageable side effects of 5‐FU alone cream or ointment under occlusion, the treatment's potential effectiveness should not be discounted.^14^ Intralesional use of 2% zinc sulfate proved beneficial in treating stubborn warts. Most likely working through necrosis and inflammation, the drug had an 81% success rate after the first injection and a 98% success rate after 2 weeks after the third injection.^15^ Bleomycin injections are placed directly into the wart. Injections are quite unpleasant and may necessitate the use of local anesthetic.^16^ More than 60% of warts cleared up after intralesional injections of 5‐fluorouracil at a dose of 40 mg/mL once weekly until 4 weeks.^17^ When taken orally, zinc may stimulate the immune system and cause warts to fall off. Research conducted in Iraq found that patients with resistant warts responded well to oral zinc sulfate 10 mg/kg/day up to 600 mg in three separate doses over the course of 1–2 months. The success rate in curing patients was 89%. The adverse effects included vomiting, nausea, and epigastric discomfort, and the mechanism of action was either the restoration of normal zinc levels or the stimulation of the immune system through its immunomodulating activity.^15^ Vitamin D, which is fat soluble, acts on the endocrine, autocrine, and paracrine systems. Vitamin D's endocrine functions typically include maintaining normal serum calcium levels. Cell types that express vitamin D nuclear receptors are the target of vitamin D's unique autocrine and paracrine activities. Potential effects include inducing cell differentiation, suppressing cell proliferation, and causing apoptosis, all of which may play important roles in immunology, cancer, and many organ systems.^18^

Modifying antigen presentation by dendritic cells (DC) or macrophages is one potential mechanism by which vitamin D influences the innate immune response to pathogens. Treatment with calcitriol has been demonstrated to decrease antigen presentation, restrain DC maturation, and mediate a tolerogenic T‐cell response, suggesting that these cells have vitamin D receptors (VDRs).^19^ Calcitriol inhibits cell proliferation and regulates cytokine synthesis, and T‐helper cells appear to be a primary target.^20^ Calcitriol suppresses Th1 cytokines and stimulates Th2 cytokines in vitro.^21^ Vitamin D also affects IL‐17‐secreting T cells (Th17 cells). In addition to helper T cells, calcitriol is a powerful inducer of regulator T cells (Tregs). Recent research has highlighted the role of regulatory T cells (Tregs) in mediating vitamin D's immunoregulatory effects.^22^ The objective of this research was to examine the effect of intralesional vitamin D3 in the management of cutaneous warts.

## 2 PATIENTS AND METHODS

The research was conducted between April 2021 and March 2022 in the dermatology department of Al‐Sadr Medical City in the city of Al‐Najaf Al‐Ashraf.

### 2.1 Ethical review board

The Arab Board of Dermatology's scientific council gave their authorization before the study could proceed. The patients and their parents were given comprehensive information about the study, including the treatment modality and duration of participation. All patients gave their informed consent about pronunciation before taking part in the trial.

The purpose of this research is to determine whether or not the intralesional vitamin D3 is useful in the treatment of skin warts.

### 2.2 Selecting a patient

Fifty people, including 21 males and 29 females, participated in the study. They lost by a count of 10 because the treatment was too disruptive to their productivity. In total, 204 cutaneous wart lesions were treated across 40 individuals (14 males and 26 females) in this randomized controlled experiment. Patients’ ages ranged from 20 months to 52 years. Patients were included who had single or multiple cutaneous warts matching the diagnostic criteria. Women who are pregnant are not eligible. Immunosuppressed patients (related to systemic corticosteroids, chemotherapy, cancer, transplant patients, etc.). Patients had utilized non‐study treatments within the month prior to the study.

At the first appointment, patients and their guardians were asked detailed questions about their medical history, including, but not limited to, name, age, sex, occupation, financial status, disease duration, lesion location, lesion count, single versus multiple lesion status, and whether or not they had previously received treatment for their warts. Prior treatment with corticosteroids or other immune suppressants, a history of warts in the family, or a diagnosis of immune suppression, organ transplant, or chronic disease should be considered.

Patients who met the inclusion and exclusion criteria underwent a thorough physical examination to determine the location, size, type, and number of lesions. Every patient was given a set of color photos taken at the start and before each treatment.

### 2.3 Treatment

**FIGURE 1:**
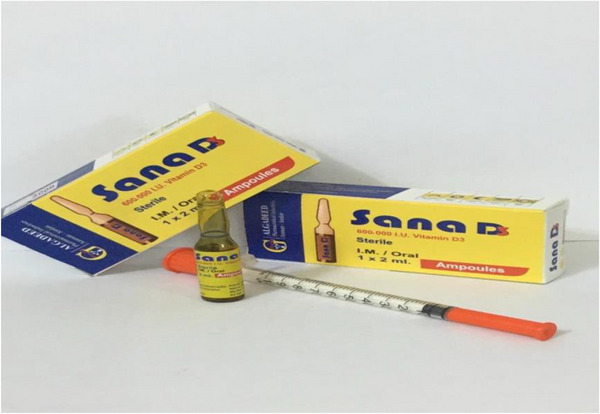
Vitamin D3 ampoules (600 000 IU).

Since the first session of vitamin D3 injections without Anastasia caused pain for all patients, 0.2 mL of lignocaine (20 mg/mL) was injected into the base of each wart slowly using a gage 27 needle. This was followed by the slow injection of 0.2 mL of vitamin D3 solution (600 000 IU; 15 mg/mL) (Figure 1). In certain cases, as many as five warts might be injected during a single session. The injections were repeated every 4 weeks until the condition improved significantly or no longer required treatment. Patients were instructed to refrain from using any external or internal wart remedies following treatment.

Lesions were measured before and after therapy to record how well the lesions responded to the treatment. To detect any recurrence, the patients were monitored for a full 6 months after their final injection. Before and after measurements of all cutaneous warts were taken during treatment in order to evaluate the efficacy of the procedure. Photographic measurements were taken at the beginning, after 8 weeks, and after 6 months for the clinical evaluation. Patients were considered to have a partial response if they experienced a 50% or higher reduction in the size and quantity of warts and to have not responded if they experienced a decrease of less than 50%.

### 2.4 Quantitative research

For this study, data from 40 individuals with cutaneous warts were input and analyzed using SPSS V.25 (SPSS 25). In the case of continuous variables like age, duration, and size of warts, descriptive statistics such as mean and standard deviation are generated. All of the percentages and frequency count for the category variables. Variables like wart size can be compared across treatment sessions using the analysis of variance, and the average duration of warts can be compared among categories of response to treatment using the same method (complete, partial, and none). The chi‐square statistic is used to examine the significance of differences between two sets of categorical data. The correlation coefficient (*R*) value was determined using bivariate correlation tests, with Pearson's correlation test being used to assess the relationship among response rates and age, number of warts, and duration of warts, and Spearman's correlation test being used to assess the relationship between gender and response rates. In contrast, an *R* number above 0.8 suggests a moderate degree of connection, whereas an *R* value below 0.4 shows a weak degree of correlation. Microsoft Word and Excel for Windows version 2016 were used to create the tables and figures, and descriptive paragraphs were included for each.

## 3 RESULTS

**FIGURE 2:**
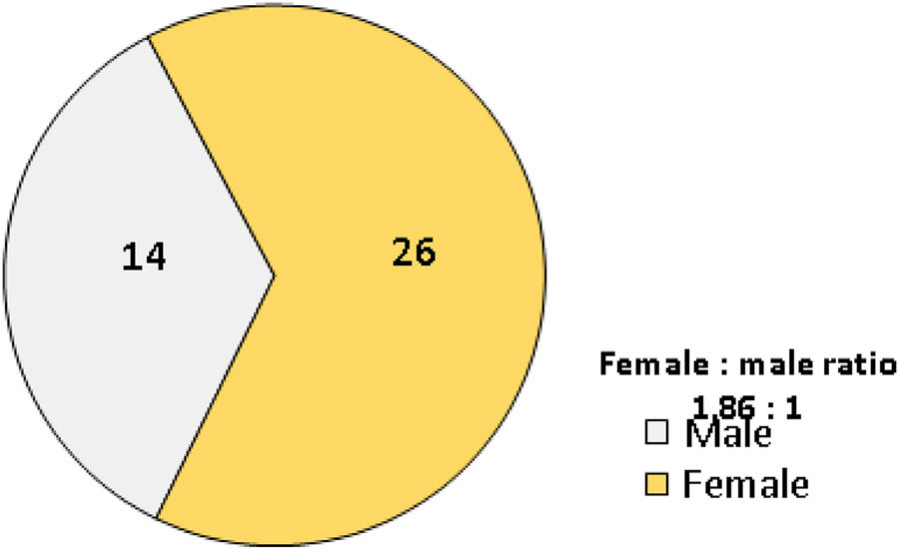
Pie‐chart showing the gender distribution and male to female ratio of 40 patients with cutaneous warts.

The average age of the 40 patients in this study was 12.8‐20.1 (range: 20 months–52years) old, whereas the median age was 18, and the interquartile range was 10–27. There were 1.86 times as many women as there were men involved in the cases. Warts often lasted anywhere from 4 months to 3 years on average (Table 1 and Figure 2). There were a total of 204 cutaneous warts among the study population, as evidenced by the frequency distribution (Table 2).

**TABLE 1:** Demographic characteristics of 40 patients with cutaneous warts.

Variable		No. of patients	%
Age (year)	≤10	11	27.5
	11–20	14	35.0
	21–30	7	17.5
	>30	8	20.0
	Total	40	100.0
	Mean (SD*)	20.1 (12.8)	–
	*Range*	1.8–52	–
Sex	Male	14	35.0
	Female	26	65.0
	Total	40	100.0
Duration of warts	*Mean (SD) (year)*	1.4 (0.8)	–
	*Range*	4 months–3 years	–

Abrreviation: SD, standard deviation.

**TABLE 2:** Frequency distribution of a number of cutaneous warts among the studied group^a^.

Number of lesions	No.	%
1	17	42.5
2–5	11	27.5
6–10	9	22.5
>10	3	7.5
Total	40	100.0

aTotal number of lesions =204.

**FIGURE 3:**
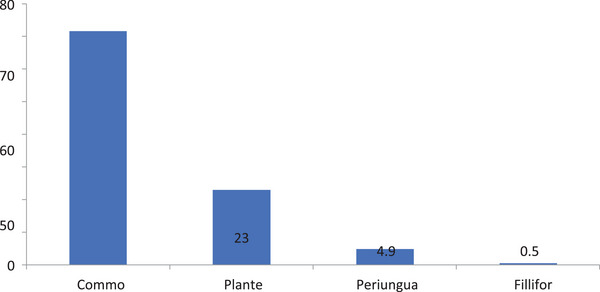
Proportional distribution of 204 warts of the 40 patients with cutaneous warts.

The majority (146/71.6%) of the 204 warts were common warts; other forms included plantar warts (23%), periungual warts (9%), and a single filiform wart (0.5%) (Table 3 and Figure 3).

**TABLE 3:** Frequency distribution of types of warts (*N* = 204).

Type of warts	No.	%
Common	146	71.6
Plantar	47	23
Periungual	10	4.9
Filiform	1	0.5
Total	204	100

**FIGURE 4:**
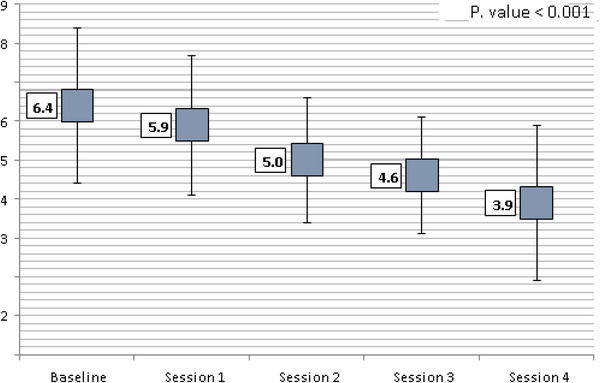
Box‐plot showing the change in mean wart size across subsequent treatment sessions. A highly significant reduction in mean size was found (*p* value <0.001).

The mean size of the 204 warts was reduced from 6.4 mm at the beginning of therapy to 3.9 mm by the end of treatment, a decrease that was statistically significant (P0.001). The trend of the reduction in mean size at succeeding sessions is shown in Figure 4. Complete resolution was seen in 167/204 (81.9%) warts, partial resolution was shown in 23/11.3% warts, and no resolution was seen in 14/6.9% warts after the fourth treatment session, as described in the study (Table 4). Table 5 also summarizes the total number of sessions and the complete response rates at each stage: 12.7% after the first session, 29.9% after the second, 54.9% after the third, and 81.9% after the fourth.

**TABLE 4:** Overall responses to treatment of 204 warts.

Response	No. of warts	%
Complete	167	81.9
Partial	23	11.3
None	14	6.9
Total	204	100.0

**TABLE 5:** Cumulative frequency and rates of complete response to treatment for 204 cutaneous warts at subsequent sessions of treatment.

	Complete response		
Session of treatment	Frequency	Cumulative frequency	Cumulative rate (%)
Session 1	26	26	12.7
Session 2	35	61	29.9
Session 3	51	112	54.9
Session 4	55	167	81.9
	167	–	–

**FIGURE 5:**
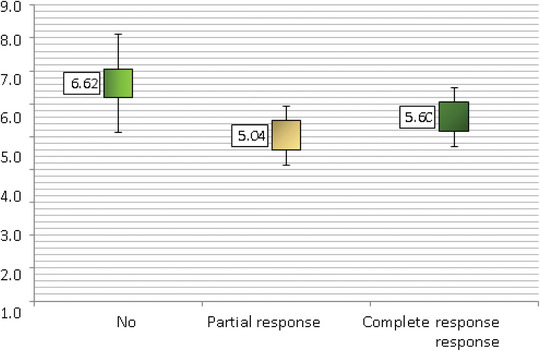
Comparison of mean size of warts across the response to treatment.

**FIGURE 6:**
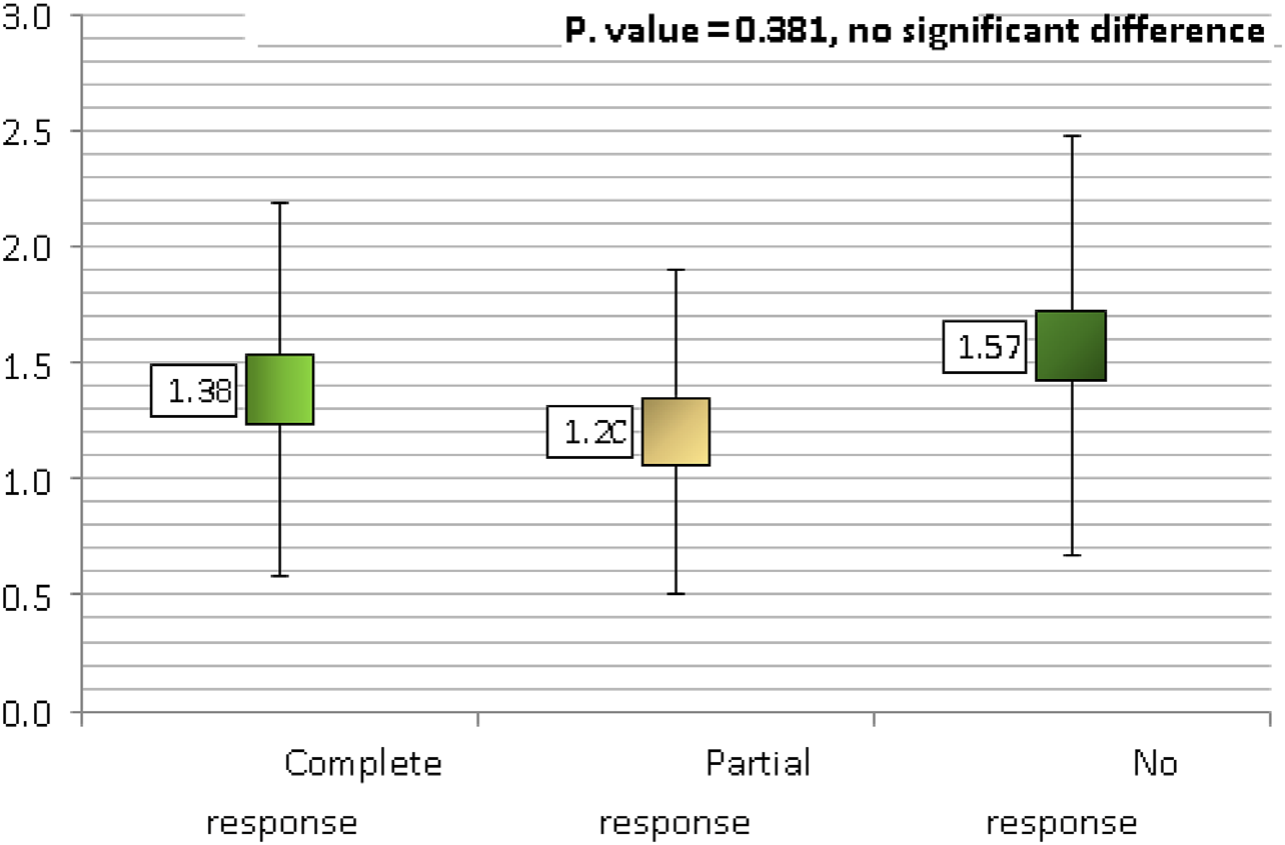
Comparison of mean duration of warts across the response to treatment.

**FIGURE 7:**
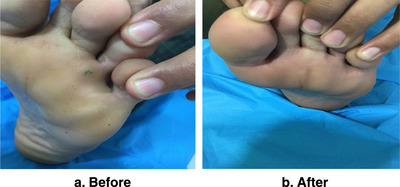
A boy of 20 years old with single plantar wart in left foot for 10 months shows a complete clearance of wart: (a) before treatment, (b) 2 months after four treatment sessions.

**FIGURE 8:**
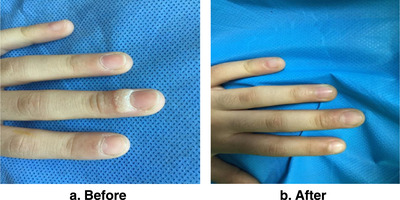
A girl of 9 years old with single wart in left third periungual for 1 year shows complete clearance: (a) before treatment, (b) 1 month after two treatment sessions.

**FIGURE 9:**
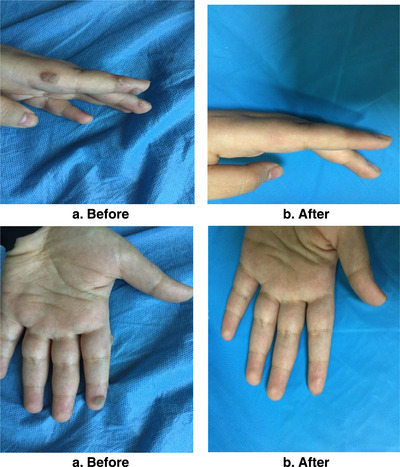
A girl of 20 years old with multiple common warts in hand shows complete response: (a) before treatment, (b) 6 weeks after three treatment sessions.

**FIGURE 10:**
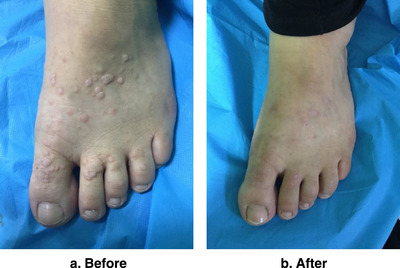
Women 38 years old with multiple warts in left dorsal foot show complete clearance: (a) before treatment, (b) 2 months after four treatment sessions.

Using cross‐tabulation, we compared patients whose warts were smaller than 5 mm in diameter to those whose warts were larger than 5 mm in diameter; we found no statistically significant correlation between wart size and treatment outcome (*p* > 0.05) (Table 6 and Figure 5). There was also a statistically significant (*p* = 0.001) correlation between the type of wart and the degree to which it responded to treatment; patients with periungual warts had a 100% complete response rate, those with plantar warts had a 91.5% complete response rate, and those with common warts showed a 77.4% complete response rate (Table 7). There were no statistically significant differences in the mean duration of warts across the response rate subgroups (complete, partial, and no response), though the none response subgroup had a longer mean duration (1.57 years) than the partial response (1.20 years) or the complete response (1.38 years) (Figure 6), (Figures 7, 8, 9, 10).

**TABLE 6:** Cross‐tabulation for the relationship between size of wart and response to treatment for 204 cutaneous warts of 40 patients.

	Size of wart				Total		
	≤5 mm		>5 mm				
Responses	No.	%	No.	%	No.	%	*p* Value (chi‐square)
Complete response	94	83.9	73	79.3	167	81.9	0.227 ns
Partial response	13	11.6	10	10.9	23	11.3	0.374 ns
No response	5	4.5	9	9.8	14	6.9	Reference group
Total	112	100.0	92	100.0	204	100.0	

**TABLE 7:** Cross‐tabulation for the relationship between type of wart and response to treatment for 204 cutaneous warts of 40 patients.

	Responses								
	Complete response		Partial response		No response		Total		
Type of warts	No.	%	No.	%	No.	%	No.	%	*p* Value (*z*‐test)
Common	113	77.4	19	13	14	9.6	146	100.0	<0.001
Plantar	43	91.5	4	8.5	0	0.0	47	100.0	<0.001
Periungual	10	100.0	0	0.0	0	0.0	10	100.0	<0.001
Filiform^a^	1	100.0	0	0.0	0	0.0	1	100.0	–
Total	167	81.9	23	11.3	14	6.9	204	100.0	

^a^ Filiform wart was excluded from the comparison.

Bivariate Pearson's and Spearman's correlation tests were employed to investigate the relationship among age, gender, number of warts, and length of warts and response to therapy, and both tests found no significant association between these factors (*p* value >0.05) (Table 8).

**TABLE 8:** Results of bivariate correlation analysis for the response to treatment and each of age, gender, number, and duration of warts for 40 patients with cutaneous warts.

	Parameter of correlation with response to treatment^a^	
Variable	Correlation coefficient (*R*)	*p* Value
Age	−0.083	0.240
Gender	0.018	0.798
Number of warts	0.054	0.742
Duration of warts	0.007	0.917

^a^ Pearson's correlation test used for age, number, and duration of warts, whereas Spearman's correlation test used for gender versus response rates.

Minor side effects, none of which were life‐threatening, were experienced by 13 patients. Seven (53.8%) patients experienced injection‐site swelling, which went down on its own within a week. Five (38.5%) patients experienced itching, and one (7.7%) patient experienced dyspigmentation, which went away on their own after 3 weeks (Table 9).

**TABLE 9:** Frequency of side effects of intralesional vitamin D3 treatment for cutaneous warts.

Side effect	No.	%
Swelling at injection site	7	53.8
Itching	5	38.5
Dyspigmentation	1	7.7
Total	13	100

## 4 DISCUSSION

To put it simply, warts are a harmless, widespread, and frequently recurring HPV infection of the skin and mucous membranes.^1^ Most people ask for treatment for warts because they are cosmetically bad appearance and occasionally painful, especially when they are located on the soles of the feet.^23^ Cryotherapy and electrocautery are just two examples of the damaging treatments that may be necessary when treating many warts at once, especially palmoplantar. Both scarring and discoloration can be the end effect of such invasive operations. Warts are unsightly, can be embarrassing, and have a high recurrence rate and resistance to common treatments. Due to its ability to increase the body's immune response against HPV, immunotherapy is currently the most effective method for treating warts. Comparatively few occurrences recur when compared to other destructive modalities.^23^ Immune therapy has been explored with a wide range of antigens and vaccines, including bleomycin, Candida albicans, PPD, MMR, and mycobacterium W vaccine.^24^

This research found that administering vitamin D3 intralesional was an effective method for treating cutaneous warts. Forty Iraqi patients (14 males and 26 females) with a total of 204 cutaneous warts were treated by injecting 0.2 mL of vitamin D3 (600 000 IU; 15 mg/mL) gently in the base of each wart lesion, with a cap of 5 lesions each session. Up to four treatments, each spaced 2 weeks apart, may be necessary to completely remove the warts. After 2 months, 167 warts (81.9%) were completely gone, 23 warts (11.3%) had a partial reaction, and 14 warts (6.7%) had not responded at all. It is unknown how vitamin D actually works to get rid of warts. Vitamin D derivatives are thought to be effective against warts because of their potential to control the growth and differentiation of epidermal cells and the secretion of cytokines. The VDR and vitamin D‐1‐hydroxylase gene expression was discovered to be elevated upon toll‐like receptor activation of the macrophages of the human, leading to the promotion of antimicrobial peptide production.^25^ Inhibiting the expressions of IL‐6, IL‐8, TNF‐alpha, and TNF‐gamma via a VDR‐dependent pathway has been shown to have immunomodulatory effects in experimental settings.^26^

Mild swelling and itching at the injection site were the only adverse events seen in our trial, and both resolved on their own within a few days. Clearance of the injected warts was accompanied by removal of warts at unrelated anatomical sites, suggesting that the immune response is systemic rather than localized to the injection site. All but one patient who initially showed only a partial response to the treatment eventually saw their warts completely disappear over the course of the 6‐month follow‐up period. As for warts, they did not come back during the follow‐up.

Vitamin D3 administered intralesional has been studied extensively for its potential to effectively treat warts. Twenty patients with multiple or single plantar warts participated in the study by AKtas et al. Patients averaged two sessions of treatment every 4 weeks. Of the total number of patients, 16 (80%) showed complete response, 1 (10%) showed partial reaction, and 3 (0%) showed no response at all.^27^ Only four of the 204 lesions studied in this investigation had only a partial response, but among the planter warts (47/204) that did respond, the complete response rate was significantly greater (91.5% vs. 68.6%).

Moscarelli et al. successfully cured a refractory wart in a 41‐year‐old patient with a history of renal transplantation with calcitriol solution.^28^ Calcipotriene ointment was shown to be effective in the total elimination of a patient's anogenital wart, as demonstrated by Rind et al.^29^ Although the vitamin D3 employed in these two case studies was applied topically (in the form of a solution or ointment), we administered it intralesional and did not include individuals who were immunocompromised.

Of the 60 patients enrolled in the study by Naresh et al., 60% had palmoplantar warts, 20% had common warts, 10% had periungual warts, and 10% had filiform warts, repeating the same regimen every 3 weeks with the same dosage and technique. Forty‐eight of the patients (80%) demonstrated a full recovery, whereas six patients (10%) had a moderate reaction, and six patients (10%) had a minor one. During a median follow‐up of 6 months, warts returned in four patients (6.66%).^30^ Warts did not return during the follow‐up phase of the current trial, even though treatments were administered every 2 weeks.

A total of 42 individuals with numerous warts participated in the Kavya research by Marjunath et al. In a study where patients only had two warts injected at a time, 78.57% saw complete clearance, 14.2% exhibited a moderate response, and 7.1% saw a mild response. Recurrence of palmoplantar warts occurred in only one patient.^31^ The current trial allowed for the treatment of up to five lesions per session.

The effectiveness of intralesional zinc sulfate 2% solution versus vitamin D3 in the treatment of plantar warts was examined in a study by El Sayed et al. A total of 105 participants participated in the study, with 35 individuals representing each of the three categories. Intralesional injection of 2% zinc sulfate solution was used in the first group, vitamin D3 was injected into the second, and intralesional injection of normal saline was utilized in the third. At most, each patient received four treatments at 2‐week intervals. Complete response was higher in the zinc group (71.4%), compared to the vitamin D3 group (62.9%), and the normal saline group (40%) at the end of the research. Intralesional injection of 2% zinc sulfate was found to be more efficacious than intralesional injection of vitamin D3 in this investigation.^32^ The present study found a response rate of 91.5% for plantar warts.

Each of the 62 participants in the study by El Taieb et al. had at least three common warts, of which some received intralesional PPD injections, others received vitamin D3 injections, and the rest served as controls. Treatment sessions occurred every other week for a maximum of four sessions. The results showed that among those who were treated with PPD, 43 patients (69.4%) saw complete clearance, whereas among those who were given vitamin D3, 22 patients (35.5%) saw complete response, and 21 patients (33.9%) saw partial response. Each set of findings was statistically significant when compared to the control group. PPD showed statistically significant superiority over vitamin D3 in the common warts treatment.^33^ One hundred and thirteen lesions (77.4%) responded completely to treatment for common warts, whereas 19 lesions (13%) did not. The efficacy of both MMR and intralesional vitamin D3 in the treatment of warts was compared in a study by Shaldoum et al. Sixty patients participated in the study, with half assigned to get intralesional MMR vaccine into the largest wart and the other half receiving intralesional vitamin D3 in up to five warts at once. Each group participated in the session no less frequently than every 3 weeks, and for no more than 6 total sessions. The numbers showed that in group A, 80% of patients were completely cured, whereas 6.67% of patients in group B experienced the same. Neither group differed from the other in any meaningful way.^34^

## 5 CONCLUSION

When it comes to treating cutaneous warts, whether they be single or many, an intralesional injection of vitamin D3 is a highly successful and inexpensive immunotherapy option.

## CONFLICT OF INTEREST STATEMENT

We declare that on one of the authors has conflict of interest.

## FUNDING INFORMATION

The funding sources for the work are at the author's personal expense.

## Data Availability

Not applicable.
